# Control of progesterone receptor transcriptional synergy by SUMOylation and deSUMOylation

**DOI:** 10.1186/1471-2199-13-10

**Published:** 2012-03-22

**Authors:** Hany A Abdel-Hafiz, Kathryn B Horwitz

**Affiliations:** 1 Division of Endocrinology, Department of Medicine, Anschutz Medical Campus, University of Colorado, Aurora, CO 80045, USA; 2 Department of Pathology, Anschutz Medical Campus, University of Colorado, Aurora, CO 80045, USA; 3 University of Colorado AMC, Mail Stop 8106, 12801 East 17th Avenue, Aurora, CO 80045, USA

## Abstract

**Background:**

Covalent modification of nuclear receptors by the Small Ubiquitin-like Modifier (SUMO) is dynamically regulated by competing conjugation/deconjugation steps that modulate their overall transcriptional activity. SUMO conjugation of progesterone receptors (PRs) at the N-terminal lysine (K) 388 residue of PR-B is hormone-dependent and suppresses PR-dependent transcription. Mutation of the SUMOylation motif promotes transcriptional synergy.

**Results:**

The present studies address mechanisms underlying this transcriptional synergy by using SUMOylation deficient PR mutants and PR specifically deSUMOylated by Sentrin-specific proteases (SENPs). We show that deSUMOylation of a small pool of receptors by catalytically competent SENPs globally modulates the cooperativity-driven transcriptional synergy between PR observed on exogenous promoters containing at least two progesterone-response elements (PRE_2_). This occurs in part by raising PR sensitivity to ligands. The C-terminal ligand binding domain of PR is required for the transcriptional stimulatory effects of N-terminal deSUMOylation, but neither a functional PR dimerization interface, nor a DNA binding domain exhibiting PR specificity, are required.

**Conclusion:**

We conclude that direct and reversible SUMOylation of a minor PR protein subpopulation tightly controls the overall transcriptional activity of the receptors at complex synthetic promoters. Transcriptional synergism controlled by SENP-dependent PR deSUMOylation is dissociable from MAPK-catalyzed receptor phosphorylation, from SRC-1 coactivation and from recruitment of histone deacetylases to promoters. This will provide more information for targeting PR as a part of hormonal therapy of breast cancer. Taken together, these data demonstrate that the SUMOylation/deSUMOylation pathway is an interesting target for therapeutic treatment of breast cancer.

## Background

Progesterone plays a key role in the development, differentiation and maintenance of normal and malignant female tissues. Its effects are mediated by progesterone receptors (PRs), members of the steroid hormone receptor superfamily of ligand-dependent transcription factors. PRs exist as two major, functionally different [1] isoforms--PR-A (~94 kDa) and PR-B (~110 kDa). They are multidomain proteins consisting of a central DNA-binding domain (DBD); large N-termini with a proximal activation function (AF-1) common to both isoforms; a distal AF-3 in the B-upstream segment (BUS) restricted to PR-B; and at their C-termini, a nuclear localization signal in a hinge region upstream of an AF-2-containing ligand binding domain (LBD) [1-5]. PRs are transactivators that can be tethered to DNA through other transcription factors [6-10] but more commonly are bound directly to DNA at palindromic progesterone-response elements (PREs) [11]. The two isoforms bind DNA with equivalent affinity [12] so this cannot explain their functional differences. Rather, dissimilar coregulator recruitment has been invoked for their differences [13]. These coactivators or corepressors facilitate receptor/DNA occupancy, chromatin remodeling and recruitment of general transcription factors associated with the RNA polymerase II holocomplex [14]. Function of the receptors and their coregulators are in turn controlled by posttranslational modifications including phosphorylation, acetylation, ubiquitination and SUMOylation [15] that influence hormone sensitivity and promoter selectivity, among others [16]. Ubiquitination for example, promotes ligand-dependent PR protein downregulation *via *proteasomal degradation, which paradoxically maximizes transcriptional activity [17]. Because these modifications are reversible, enzymes that dephosphorylate, deacetylate, deubiquitinate and deSUMOylate PRs also alter activity [16,18-20], so that permutations of these modifications undoubtedly play a large role in the complex signaling patterns ascribed to the receptors [1].

### Transcriptional synergy and PR SUMOylation

Additional complexity arises from the structure of DNA to which PRs bind. Cooperativity among receptors bound at compound promoters consisting of two or more PREs results in synergism defined as a "more-than-additive" transcriptional effect [21]. Iniguez-Lluhi and Pearce [21] first identified a short synergy control (SC) motif in glucocorticoid receptors (GR) that disrupted synergy on promoters with multiple response elements. Its mutation induced strong synergistic effects but only at compound response elements. The SC motif turned out to be a SUMOylation site at which conjugation of SUMO-1, a 97 amino acid (aa) Small Ubiquitin-like Modifier, disrupted synergy [22-24]. Similar sites in both GR and PR [15] contain a lysine (Lys, K) residue embedded in the consensus sequence ΨKxE (where Ψ is a large hydrophobic amino acid, and x is any amino acid) located in the N-terminal AF-1 domains of the receptors. For human PR-B this sequence is centered at K388, and at a homologous site of PR-A. Monomeric SUMO-1 covalently binds this site through a series of dynamic and reversible enzymatic reactions involving an E1 SUMO activating enzyme, an E2 conjugating enzyme (Ubc9) and E3 ligases (PIASs; Protein Inhibitors of Activated STAT (Signal Transducer and activator of transcription)). DeSUMOylation is catalyzed by one of six human Sentrin-specific proteases (SENPs) that target SUMO. Largely due to their roles in modifying the activity of steroid receptors, both Ubc9 and PIAS have at times been classified as transcriptional coregulators [25-27]. Mouse knockouts of Ubc9 or SENP1 are embryonic lethal, demonstrating that the balance of SUMOylation and deSUMOylation is essential for development [28,29]. Most, but not all steroid receptors--the exception appearing to be estrogen receptors (ER)--are targets of SUMOylation. This is consistent with the fact that phylogenetic and sequence alignments of GR, mineralocorticoid receptors (MR), androgen receptors (AR) and PR links them to a steroid receptor subfamily characterized by much larger N-termini (ranging from 420 to 602 aa) than the N-termini of ERα or ERβ (184 and 148 aa, respectively). As a result *in vitro *translated AR and GR, but not ERα or ERβ, are SUMOylated [30].

SUMO conjugation of PR-B at K388 (or the PR-A equivalent) is hormone dependent and occurs via PIAS1 [31] or PIAS3 [32]. This suppresses PR -dependent transcription of promoters containing multiple PREs but not a single PRE [6,18,33-35]. Additionally, overexpression of PIAS3 can induce PR-B SUMOylation at K7 and K531 [32] but the physiological relevance of this is unclear. SUMO is deconjugated from the receptors by SENPs, which, like deSUMOylation by mutation of K388, dramatically enhances PR transcriptional activity [18]. The relationship between the transcriptional efficacy of deSUMOylation and the role of ligand-dependent PR downregulation are contradictory. Zhang and coworker [36] showed that mutation of PR-B at K388 retards progesterone-induced degradation through the ubiquitin-proteasome pathway. In contrast, we and others [6,18] have shown that PR K388R mutants undergo accelerated ligand-dependent downregulation thereby explaining their heightened transcriptional activity.

In the present study we analyze the functional effects of SENP-induced PR deSUMOylation in detail. Our results indicate that on a compound promoter, SENP1 enhances transcription in a dose-dependent manner, but this requires full-length PR. However enhanced transcription is independent of PR DNA binding specificity or the PR S294 phosphorylation site. By deSUMOylating PR, SENP increases PR sensitivity to hormone. The histone deacetylase inhibitor Trichostatin A (TSA) has a marked biphasic effect. At high concentrations, which promote global histone hyperacetylation and modify many proteins [37], TSA strongly suppresses transcription and this is reversed by the coactivator SRC-1. However, low TSA concentrations upregulate PR-dependent transcription. This effect of TSA is uncoupled from inhibition by SUMOylation indicating that HDAC activity is not involved in transcriptional synergy controlled by SENP1.

## Results

### SENP and PR deSUMOylation

#### SUMOylation and the promoter context of PR transcriptional synergy

**Figure 1:**
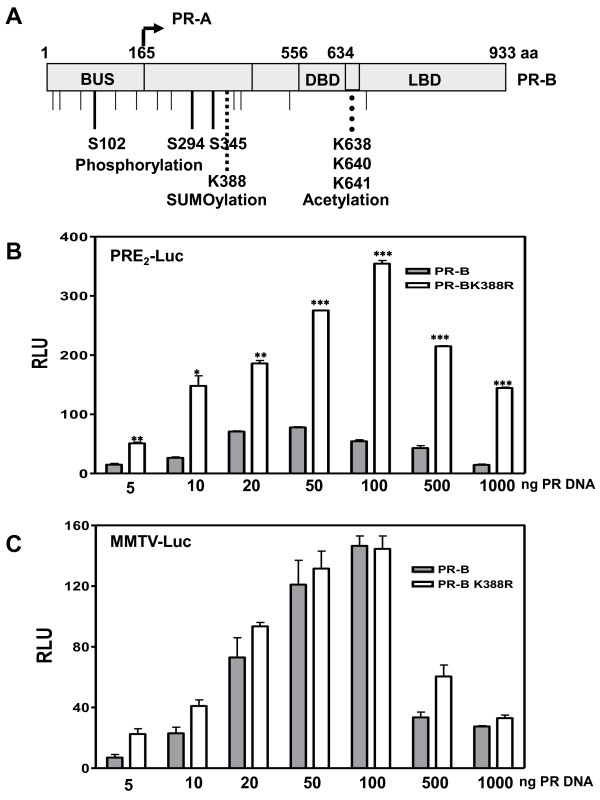
**Modulation of PR transcriptional activity by SUMO-1 depends on the promoter context**. **A) **schematic of PR-A and PR-B showing the location of hormone dependent phosphorylation sites; the Lys-388 SUMO conjugation site within an IKEE motif; and an acetylation consensus KxKK site (amino acids 638-641). BUS, B-upstream segment; DBD, DNA binding domain; LBD, ligand binding domain. **B) **HeLa cells were transfected with 2 μg of PRE2-Luc reporter, or **C) **MMTV-Luc, together with 5-1000 ng of wild-type PR-B or mutant PR-B K388R expression vectors and Renilla-Luc as an internal control. The cells were treated 24 hrs with 10 nM R5020, then harvested and lysed. The extracts were assayed for luciferase activities. Luciferase activity is expressed in relative light units (RLU). Data represent triplicates (± SD). Statistical significance was computed by unpaired student's *t *test. *p < 0.05.

Figure 1A is a schematic of PR-B and PR-A showing location of the single ψKxE SUMO-conjugation motif centered at K388 of PR-B (and a homologous K224 of PR-A). Also shown are 3 hormone dependent serine (S102, S294 and S345) and multiple other (thin lines) N-terminal phosphorylation sites, and a hinge domain KxKK (aa 638-641) acetylation site.

We previously showed [6] that SUMOylation at K388 (or K224) is hormone-dependent and suppresses PR-B and PR-A-regulated transcription of an exogenous promoter containing two (PRE_2_) or more palindromic PREs but not a single PRE. To assess the generality of this, we used the MMTV-LTR, which contains 1 palindromic PRE and 3 PRE half-sites. In contrast to GRs that prefer the palindrome, the half-sites are preferentially used by PRs [38], possibly as monomers [39]. To examine the role of PR SUMOylation on transcriptional synergy involving PRE half-sites, HeLa cells were transfected with 5-1000 ng of DNA encoding wild-type PR-B or the SUMOylation-deficient K388R PR-B mutant [33], together with the PRE_2_-Luc (Figure 1B) or MMTV-Luc (Figure 1C) reporters, in the presence of the progestin R5020. PR-B were tested since they are more potent transactivators of the MMTV-LTR than PR-A [40]. On PRE_2_-Luc, wild-type PR-B were transcriptionally active, and mutation of their K388 SUMOylation motif synergistically raised transcription further as receptor concentrations were increased between 5 and 100 ng DNA. High PR concentrations (500 and 1000 ng DNA) led to a decrement in transcription likely due to transcription factor "squelching" [33]. Wild-type PR-B dependent transcription on MMTV-LTR showed a similar dose-dependent increase. However, absolutely no transcriptional synergy was observed with the K388R PR-B mutant suggesting that SUMOylation does not control synergy on PRE half-sites. Most of the studies below use PRE_2_-Luc

#### DeSUMOylation by SENP

**Figure 2:**
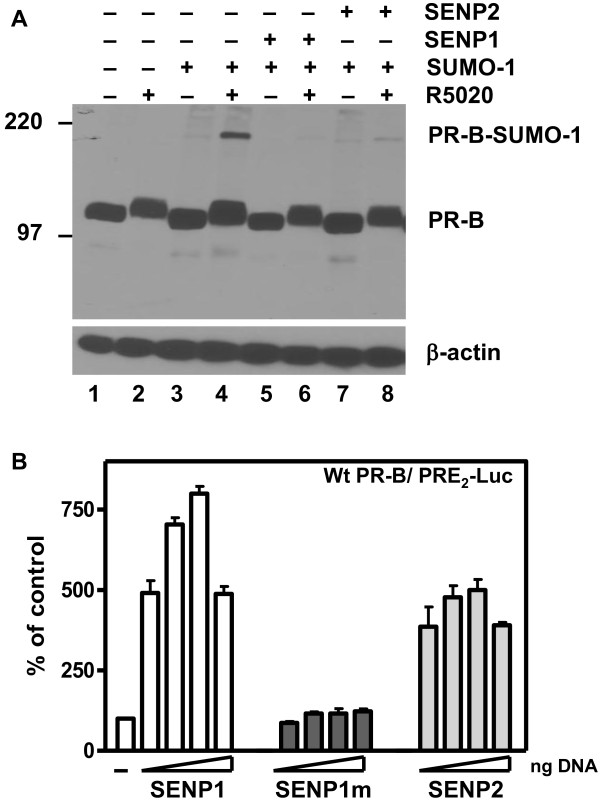
**SENP1 and SENP2 deSUMOylate PR-B and enhance their transcriptional activity**. **A) **DeSUMOylation of PR-B by wt SENP1 and SENP2. HeLa cells were cotransfected with pSG5-PR-B, GFP-SUMO-1 and SENP1 or SENP2 as indicated. Cells were grown in the presence (+) or absence (-) of R5020. PR in cell extracts separated on SDS-PAGE, were detected with anti-PR 1294 monoclonal antibody. β-actin served as a loading control. **B) **HeLa cells were transfected with the PRE2-Luc reporter plasmid in the presence of pSV40-Renilla as internal control along with PR-B and increasing amounts (50-1000 ng) of SENP1, SENP1 mutant, or SENP2 expression vectors, or an empty vector control (-). Cells were treated without (-) or with (+) 10 nM R5020 for 24 hrs before being assayed for luciferase activity. The relative luciferase activity of wt PR-B in the presence of 10 nM R5020 is set as 100%.

The K388R PR mutant is an artificial construct while proteins are naturally deSUMOylated by SENPs *in vivo *[18]. To examine effects of *in vivo *PR deSUMOylation, wild-type PR-B (1 μg DNA) and GFP-SUMO1 (0.1 μg DNA) were cotransfected into HeLa cells together with SENP1 or SENP2 expression vectors (0.1 μg DNA), and unliganded or liganded PR-B SUMOylation states were assessed by immunoblotting (Figure 2A). PR-B are not SUMOylated by ligand in the absence of SUMO-1 (lane 2), or by SUMO-1 in the absence of ligand (lane 3), but approximately 5% of the receptors are SUMOylated when both are present (lane 4). However, in cells co-expressing SENP1 (lane 6) or SENP2 (lane 8) SUMO1-PR conjugates are essentially absent. A R630L, K631M SENP1 mutant (SENP1m), whose catalytic function is disabled [20], was unable to deSUMOylate PR (Additional file 1: Figure S1A).

We next tested effects of increasing concentrations of DNA (20-1000 ng) encoding SENP1, SENP1m and SENP2 on PRE_2_-Luc transcription by R5020-liganded, wild-type PR-B transiently expressed in HeLa cells (Figure 2B) or stably expressed in T47D breast cancer cells (Additional file 1: Figure S1B). Analogous to the K388R SUMOylation-deficient PR-B mutant, deSUMOylation by SENP1 and SENP2 strongly enhanced the transcriptional activity of wild-type liganded PR-B in both cell types in a dose-dependent manner. The SENP1m control was ineffective (Figure 2B). It is of interest that these extensive transcriptional effects of SUMOylation/deSUMOylation are regulated by a minor subpopulation of PR molecules (Figure 2A). Indeed, the PR SUMOylation state and its control of transcription applies even to weak progestin agonists as shown by the fact that deSUMOylation by SENPs intensifies transcription by the mixed agonist/antagonist RU486 [41], but has no effect on transcription by the pure antagonist ZK98299 (Additional file 1: Figure S1C

**Figure 3:**
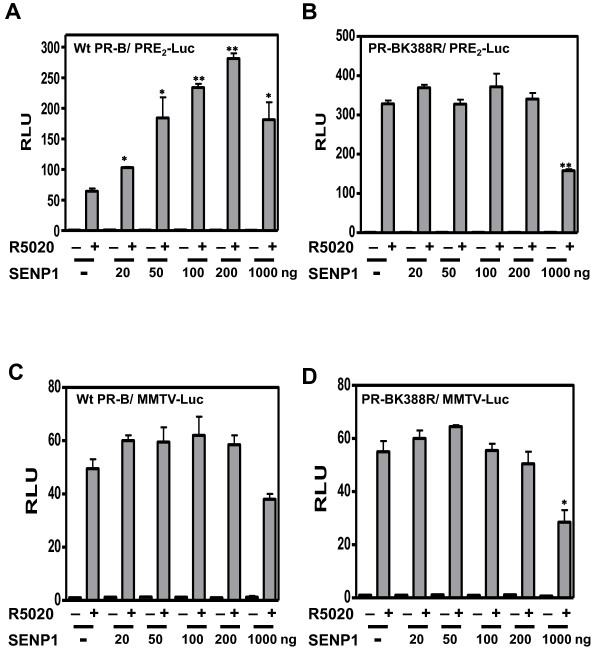
**The enhancement of PR transcriptional activity by SENP1 depends on an intact SUMO conjugation site**. HeLa cells were transfected with the PRE2-Luc (**A **and **B**) or MMTV-Luc (**C **and **D**) reporter plasmids in the presence of pSV40-Renilla as internal control along with PR-B (**A **and **C**) or PR-B K388R (**B **and **D**) expressing vectors, and a Flag-SENP1 expression vector at doses of 20, 50, 100, 200 and 1000 ng of DNA or an empty vector control (-). Cells were treated without (-) or with (+) 10 nM R5020 for 24 hrs before being assayed for luciferase activity as in Figure 1. RLU of wt PR-B in the absence of hormone is set as 1. Statistical significance was computed by unpaired student's *t *test. *p < 0.05.

The above results indicate that the activity of agonist-occupied PRs can be regulated dynamically and reversibly by SUMOylation/deSUMOylation of a small receptor subpopulation. To demonstrate whether this is a direct effect on PRs, or an indirect effect on SUMOylation of coregulators brought to the transcription complex by PRs, wt PR-B (Figure 3A, C) or the PR-B K388R mutant (Figure 3B, D) were co-expressed with increasing (20-1000 ng) concentrations of SENP1, and tested on PRE_2_-Luc (Figure 3A, B) or MMTV-Luc (Figure 3C, D). SENP1 enhanced PR-B-dependent transcription in a dose-dependent manner on PRE_2_-Luc, but was ineffective in modifying transcription by PR-B K388R on the same reporter, indicating that the response to SENP1 requires the PR SUMOylation site. This was confirmed on MMTV-Luc (or a single PRE, not shown) where SENP1 had no effect despite strong transcription with wild-type PR-B, confirming that the PREs of MMTV-LTR are not PR SUMOylation sensitive (Figure 1). We conclude that SENP1 modifies PR-dependent transcription directly at the PR SUMOylation site, which is also required for the cooperativity-driven synergy observed on a PRE_2_.

### SENP action on PR: Mechanisms

#### Activation functions

**Figure 4:**
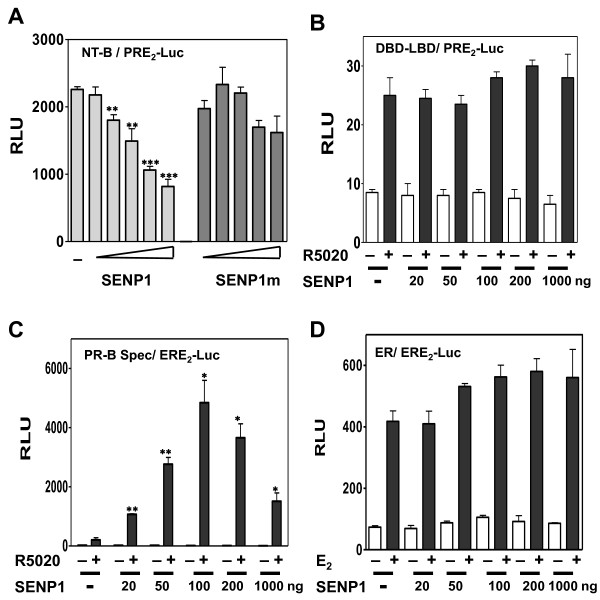
**The enhancement effect of SENP-1 on PR transcriptional activity requires full-length PR-B but not the PR DBD**. HeLa cells were transfected with the PRE2-Luc (**A **and **B**) or ERE2-Luc (**C **and **D**) reporter plasmids in the presence of pSV40-Renilla as internal control along with NT-B (**A**), PR DBD-LBD (**B**), a PR-B specificity mutant containing the ER DBD (**C**) or wild-type ER (**D**) expression vectors, and SENP1 or SENP1m (**A**, right panel) expression vectors at doses of 20, 50, 100, 200 and 1000 ng of DNA or an empty vector control (-). Cells were treated without (-) or with (+) 10 nM R5020 (**A **and **C**) or 1 nM 17β-estradiol (E2) (**D**) for 24 hrs before being assayed for luciferase activity. The values are expressed as relative luciferase units normalized to Renilla controls. Statistical significance was computed by unpaired student's *t *test. *p < 0.05.

To assess whether SENP modifies activity *via *AFs, two PR deletion mutants were tested: 1) NT-B, a constitutively active PR N-terminal construct [42] containing AF-3, AF-1 and its ψKxE SUMOylation site, linked to the DBD but missing the C-terminal AF-2 of the LBD (Figure 4A); 2) DBD-LBD, the PR DBD linked to the C-terminal LBD and its AF-2 (Figure 4B). The constructs (100 ng DNA) were transfected into HeLa cells expressing increasing concentrations (20-1000 ng) of DNA encoding SENP1 or SENP1m and transcription was measured using PRE_2_-Luc. NT-B is strongly active in the absence of ligand (Figure 4A). Despite containing the PR SUMOylation site, SENP1 was unable to further increase this strong constitutive activity. This confirms that NT-B is not SUMOylated in the absence of the LBD [33], making it insensitive to SENP1. Rather, we observe a dose-dependent repression by SENP1 requiring its catalytic activity (compare SENP1 vs. SENP1m) suggesting an effect by SENP1 on deSUMOylation of N-terminal interacting coregulatory factors. Wild-type SENP1 does not have a repressive effect on the weak ligand-dependent transcription of DBD-LBD (Figure 4B); likely the target of different, possibly non-SUMOylated, C-terminal interacting coregulators.

#### DNA binding specificity

Next we assessed the role of the PR DBD in mediating effects of SENP1 using two additional constructs: 1) a full-length PR-B Spec "specificity" mutant in which the PR DBD was replaced by the DBD of ER (Figure 4C), and 2) wild-type ER (Figure 4D). Both were tested on tandem estrogen response elements (ERE_2_) linked to luciferase. The PR-B specificity mutant was treated with R5020 (since it contains a PR LBD); ER was treated with 17β-estradiol. The receptor-encoding constructs (100 ng DNA) were transfected into HeLa cells without or with hormones together with increasing (20-1000 ng) SENP1 concentrations. The PR-B specificity mutant exhibited weak ligand-dependent transcriptional activity, which was dramatically enhanced by SENP1-mediated deSUMOylation in a dose-dependent manner. This suggests that unlike the PR LBD, neither the PR DBD nor its DNA binding site influence SUMOylation of the PR N-terminus. The DBD dimer interface of steroid receptors stabilizes binding to palindromic HREs. Interestingly, disruption of the dimer interface markedly increases transcriptional activity of receptors bound to multiple PREs (Additional file 1: Figure S2) indicating that DBD dimerization generally suppresses synergy. Wild-type ERs were unaffected by SENP1, consistent with our previous report [33] that ERs are not substrates of SUMOylation. This failure is not controlled by the ER DBD or EREs since both support SUMOylation in the context of PR-B. Unlike N-terminal coregulatory proteins of PR (Figure 4A), ER transcriptional coregulators appear to be unaffected by their SUMOylation state.

#### Sensitivity to ligand

**Figure 5:**
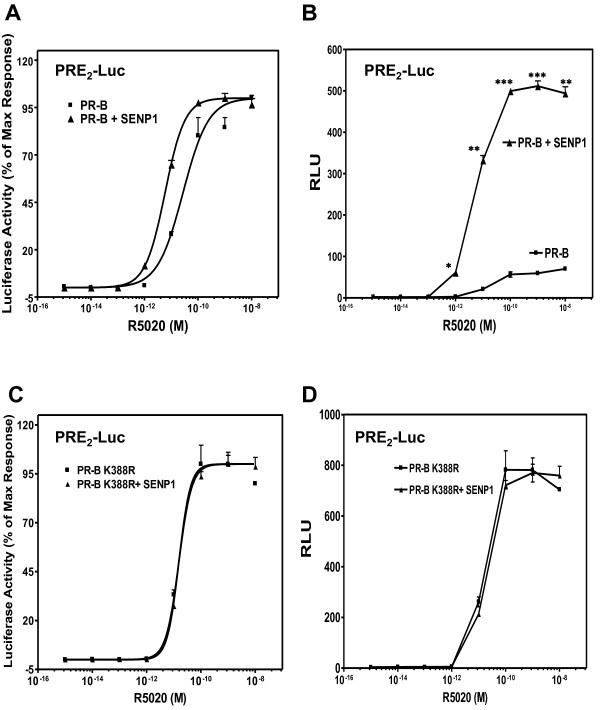
**Overexpression of SENP1 raises PR-B sensitivity to hormone**. HeLa cells were transfected with 50 ng of the PR-B (**A**) or PR-B K388R (**C**) expression vectors, a PRE2-luc, and Renilla control plasmid in the presence or absence of 100 ng of SENP1 expression vector and treated with ethanol or various concentrations of R5020 for 24 h. The average was plotted as a percentage of the maximal induction by 10 nM R5020 above no hormone levels. Curve fitting was performed by Prism Graph as described under "Experimental Procedures". The S.D. of triplicate values is indicated by the error bars. The corresponding relative luciferase activities were plotted for PR-B (**B**) and PR-B K388R (**D**). Statistical significance was computed by unpaired student's *t *test. *p < 0.05.

Since SUMOylation reduces PR-B sensitivity to hormone [6,18] we speculated that deSUMOylation by SENP would reverse this effect. To test this, HeLa cells expressing constant levels of PR-B or the PRB K388R mutant (50 ng DNA), in the absence or presence of constant SENP1 levels (100 ng DNA) were treated 24 hrs with R5020 at doses ranging from 10^-15 ^to 10^-8 ^M. Transcription levels on PRE_2_-Luc were plotted as a percent of maximal induction by 10^-8 ^M R5020 above "no hormone" controls. Curve fitting was performed by Prism Graph as described under "Experimental Procedures" (Figure 5). SENP1 reduced the dose of R5020 required for half-maximal transcription (EC50) by wild-type PR-B ~4.7-fold, from 2.74^-11 ^M to 5.85^-12 ^M (Figure 5A). SENP1 had little or no effect on the EC50 (~1.5^-11 ^M) of the SUMOylation deficient K388R mutant whose intrinsic R5020-binding affinity exceeded that of wild-type PR ~2-fold. This indicates that deSUMOylated PR are exquisitely sensitive to very low hormone concentrations; also explaining enhancement of the agonist properties of RU486 (Additional file 1: Figure S1C). Saturating hormone concentrations were similar for the two receptors (Figure 5B, D).

#### SENP, PR phosphorylation and MAPK signaling

**Figure 6:**
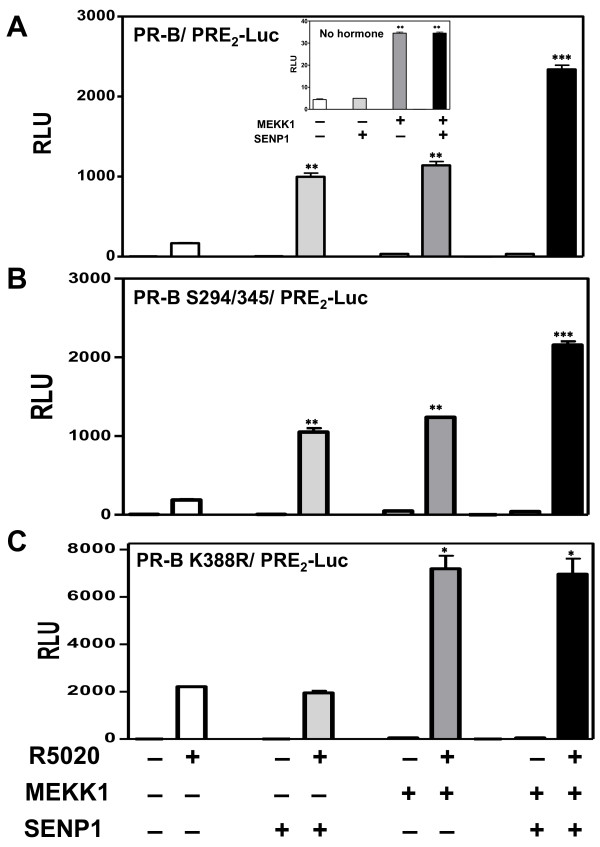
**The stimulatory effect of MEKK1 on PR-B transcriptional activity is independent of the SUMO conjugation site**. HeLa cells were transfected with 2 μg of PRE2-luciferase reporters together with 50 ng of wild type PR-B (**A**), PR-B S294/345 phosphorylation mutant (**B**), or PR-B K388R SUMOylation deficient mutant (**C**) expression vectors and Renilla-Luc as an internal control in the presence or absence of 100 ng SENP1 and/or constitutively active MEKK1 expression vectors. The cells were treated for 24 hrs with the agonist R5020 (10 nM) then harvested and lysed. The extracts were assayed for luciferase activities as in Figure 1.

PRs are phosphorylated on multiple serine residues (Figure 1), three of which--S102, S294 and S345--are currently known to be ligand-dependent [43-45]. Contradictory reports indicate on the one hand that PR-B phosphorylation is uncoupled from SUMOylation [6], and on the other that MAPK-catalyzed S294 phosphorylation antagonizes PR-B SUMOylation [18]. To assess interactions between deSUMOylation and MAPK signaling, we analyzed transcription in the presence of SENP1 (100 ng) and MAP/ERK Kinase Kinase (MEKK1; 100 ng), a strong activator of MAPK-dependent PR phosphorylation, using wild type PR-B, PR-B S294/345 phosphorylation-deficient mutants, or PR-B K388R SUMOylation-deficient mutants (Figure 6). On wild-type PR (Figure 6A), SENP1 and MEKK1 increased transcription equally, and their combined effects were additive. A key difference between the two is that SENP1 does not alter basal transcriptional activity, but MEKK raises it (Figure 6A, inset). The phosphorylation deficient mutant (Figure 6B) remained responsive to SENP1, dissociating S294/345 phosphorylation from deSUMOylation. Interestingly, MEKK1 also activated this mutant suggesting either that other PR sites, or PR coregulatory proteins, are MEKK-regulated in the S294/345-deficient receptors. Finally, SENP1 failed to hyperactivate the constitutively active K388R mutant (Figure 6C), as would be expected. However, MEKK1 was able to activate this SUMOylation-deficient PR or the constitutively active NT-B (Additional file 1: Figure S3A), uncoupling MEKK-dependent activation from PR K388 SUMOylation. Activation of MAPK signaling by overexpressing MEKK1 has complex, concentration-dependent effects on PR SUMOylation. At low concentrations, MEKK1 induces ligand-independent PR SUMOylation (Additional file 1: Figure S3B, lanes 3). At high concentrations, MEKK1 suppresses hormone-dependent PR SUMOylation (Additional file 1: Figure S3B, lanes 6&8).

#### SENP, histone deacetylases (HDAC) and SRC-1 coactivation

**Figure 7:**
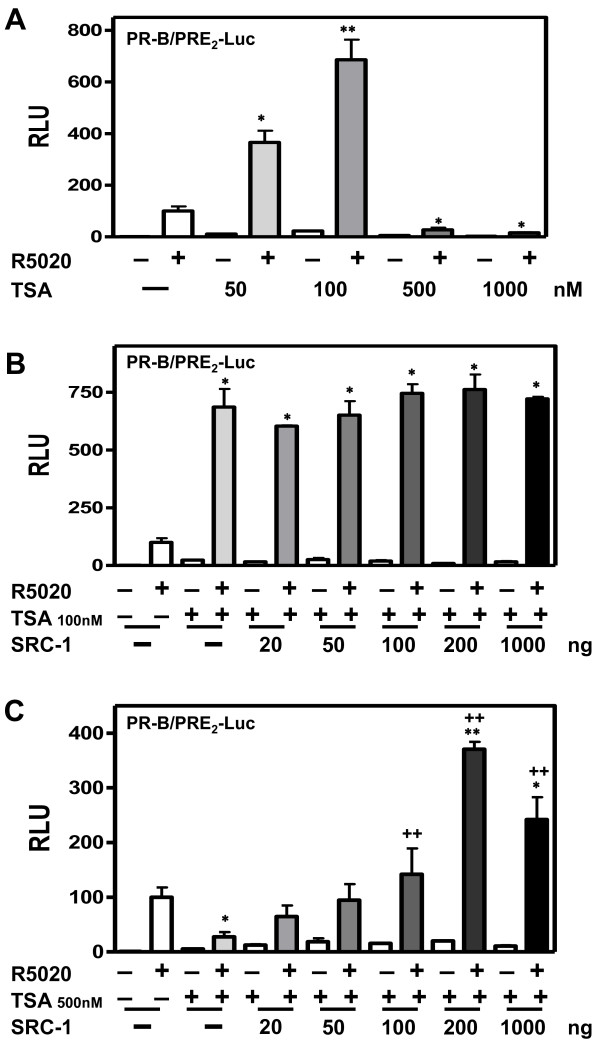
**SRC-1 reverses the inhibitory effect of the HDAC inhibitor TSA on the PR transcriptional activity**. **A**) HeLa cells were transfected with 2 μg of PRE2-luciferase reporters together with 50 ng of a PR-B and Renilla-Luc as an internal control. The cells were treated for 24 hrs with the agonist R5020 (10 nM), without (-) or with increasing amounts of trichostatin A (TSA). **B & C**) HeLa cells were transfected with 2 μg of PRE2-luciferase reporters together with 50 ng of a PR-B expression vector and Renilla-Luc as an internal control in the absence or the presence (+) of increasing amount of SRC1. The cells were treated for 24 hrs with the agonist R5020 (10 nM), without (-) or with (+) 100 nM (**B**) or 500 nM (**C**) of TSA then harvested and lysed. The extracts were assayed for luciferase activities as in Figure 1. (*) Compared with control and (+) compared with TSA treatment.

**Figure 8:**
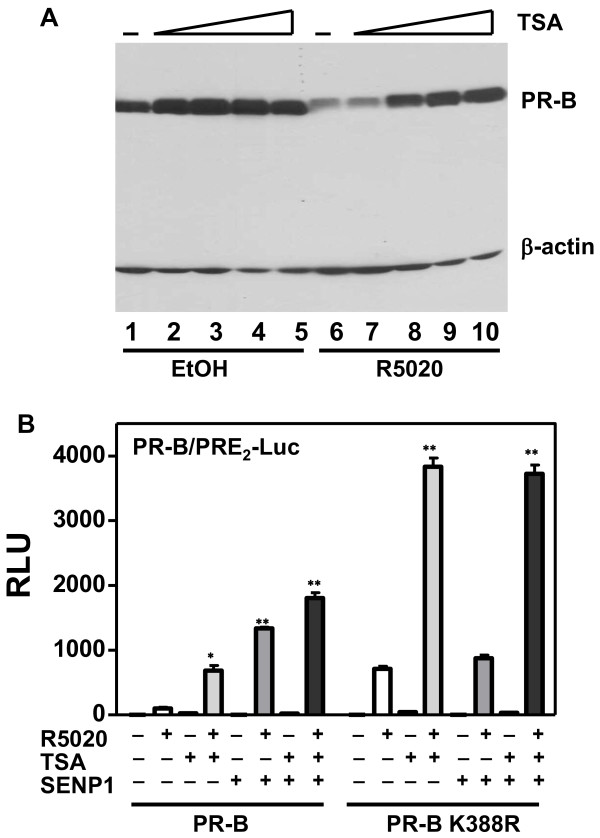
**HDACs are not a major target for SENP1 action on PR transcriptional activity**. **A) **TSA enhances PR-B protein stability. HeLa cells were transiently transfected with expression vectors encoding wild type PR-B. Cells were treated 24 hrs without (-) or with (+) 10 nM R5020 in the presence of increasing concentration of TSA. Western blot analysis was performed on cell extracts probed with the anti-PR1294 monoclonal and anti β-actin antibodies. **B) **HeLa cells were transfected with 2 μg of PRE2-luciferase reporters together with 50 ng of a PR-B (left), or the PR-B K388R mutant (right) expression vectors and Renilla-Luc as an internal control in the presence or absence of 100 ng SENP1 expression vectors. The cells were treated for 24 hrs with the agonist R5020 (10 nM), without (-) or with (+) 100 nM TSA then harvested and lysed. The extracts were assayed for luciferase activities as in Figure 1. Statistical significance was computed by unpaired student's *t *test. *p < 0.05.

Repression of the Elk-1 transcription factor by SUMOylation couples with recruitment to promoters of histone deacetylases, to further repress Elk-1 target genes [46]. This suggests that HDACs are involved in the transcriptional repression by SUMO [46]. We asked whether HDACs are involved in the synergy control and regulation of PR activity by SENP1. We first analyzed baseline effects of trichostatin A (TSA)--an HDAC inhibitor that brings about chromatin decondensation [47]--on PR-B-dependent transcription of PRE_2_-Luc (Figure 7A). There was a marked biphasic response. Compared to untreated controls, low doses of TSA (50 and 100 nM) upregulated both basal and liganded PR-B dependent transcription, while excessive TSA doses (500 and 1000 nM) were strongly inhibitory. Similar inhibitory effects of TSA have been attributed to incompatibility between hyperacetylation of chromatin and assembly of coactivators on the RNA pol II complex [48]. To assess this, we analyzed the ability of steroid receptor coactivator 1 (SRC-1) to coactivate PR-B on PRE_2_-Luc, at low (100 nM) or high (500 nM) TSA concentrations. At low TSA concentrations (Figure 7B), HeLa cells express sufficient endogenous SRC-1 to maximally coactivate PR-B dependent transcription, and exogenous addition of excess SRC-1 (20-1000 ng) does not alter these already high levels. However, high TSA concentrations (Figure 7C) repress transcription controlled by endogenous coactivators more than 90%, which exogenous SRC-1 (20-1000 ng) is able to reverse. These data support the conclusion that in HeLa cells, promoter hyperacetylation suppresses coactivator recruitment to DNA-bound PR. Additionally, we noted that high concentrations of TSA stabilize PR-B protein levels (Figure 8A; compare lane 1 vs. lanes 2-5), and prevent ligand-dependent PR-B downregulation (compare lanes 6, 7 vs. 8-10). Suppression of unliganded and/or liganded PR protein turnover would also impede transcription [49].

The relationship between HDAC inhibition and PR deSUMOylation was therefore probed using low (100 nM) TSA concentrations together with the deSUMOylase SENP1. HeLa cells were transfected with wild-type PR-B (Figure 8B, left) or the PRB K388R mutant (Figure 8B, right) in the absence (-) or presence (+) of SENP1 (100 ng) and/or TSA (100 nM). On wild-type PR-B, either TSA alone or SENP1 alone caused the expected increase in transcription. The two together were additive, suggesting a lack of interaction between them. On the K388R SUMOylation-deficient mutant, TSA was especially potent in hyperactivating the already strong basal activity. SENP1, as expected, had no effect on this basal activity. When combined with TSA, SENP1 also had no effect, suggesting that HDAC activity does not markedly contribute to transcription synergy.

## Discussion

### SUMO-dependent transcriptional repression and synergy

Various regulators of SUMO-dependent transcriptional repression have been proposed, which include chromatin-associated proteins [50], histone deacetylases [46], the SUMO-binding death-domain associated protein DAXX [51], the DEAD-box protein DP-103 [52], and the nuclear matrix protein NXP-2 [53]. The link between relief from SUMOylation and transcriptional synergy on complex promoters was first observed for GR [21,24,54,55] and later expanded to other transcription factors including the nuclear receptors AR, MR and PR [6,19,56], and transcription factors like C/EBP, SF1, MITF and ZBP89 [22,57-60]. GRs are modified post-translationally at three consensus SUMO conjugation sites, two in the N-terminus, one in the LBD [24,54,55]. Mutation of both N-terminal sites strongly enhances GR-dependent transcription on dual hormone response elements (HREs), but not on the MMTV-LTR [54]. These two N-terminal GR sites, dubbed "synergy control motifs" [21], require an intact receptor LBD and an engaged DBD dimerization interface. Holmstorm et al. [54] propose that stable binding of SUMOylated GR to multiple HREs allows recruitment of inhibitory factors, but that on non-canonical half-site elements such as the MMTV-LTR, SUMOylated GR escape these negative influences [54]. Consistent with these observations, we observe that the single N-terminal PR SUMOylation motif controls transcriptional synergy on multiple PREs but not at a single PRE [6] or the MMTV-LTR (Figure 1).

Like GR, AR are SUMOylated at two N-terminal Lys residues and mutation of one (K385) enhances cooperativity on palindromic but not direct-repeat HREs. Callevaert et al. [61] posit that this is a reflection of differing AR dimer conformations on the two types of DNA binding sites. The DBD dimer interface of steroid receptors stabilizes binding to palindromic HREs but this structure forms only after the receptors have bound to DNA [62]. This interface is essential for transcriptional activity on a single HRE, so that mutations in either MR or GR that destabilize it, disrupt receptor/DNA interactions. However, paradoxically these same dimer interface mutations markedly increase synergistic activity of receptors bound to multiple HREs while only modestly increasing DNA binding (62). Mutations in PRs that destabilize the DBD dimer interface also disrupt receptor binding and activity at a single PRE [11], while the same mutations dramatically enhance PR transcriptional activity on promoters containing multiple PREs (PR-B DX; Additional file 1: Figure S2). These mutants are still subject to SUMOylation however (data not shown), suggesting that, as previously reported for GR [54], SUMOylation is upstream of synergy control. Liu et al. (62) postulate that an inhibitory interaction between the N-terminus and the wild-type DBD dimer interface is relieved by DBD mutations, thereby promoting cooperative binding among multimeric receptors and/or coregulatory factors. We speculate that this inhibitory factor is the 97aa SUMO peptide bound at the N-terminus. Its removal, by mutation of the SUMOylation motif or enzymatically with SENP1, relieves the inhibition and permits assembly of higher order PR complexes on DNA.

### DeSUMOylation by SENP

The SENPs deconjugate SUMO-modified proteins and are critical for maintaining physiological ratios of SUMOylated to deSUMOylated substrates. Studies in knockout mice demonstrate that a fine balance of SUMOylation/deSUMOylation is required for normal embryonic development [29]. This balance may be altered in malignancies. Persistent elevation of SENP1 facilitates the transformation of the normal prostate to a dysplastic state in transgenic mice. Increased SENP expression is observed in malignancies including oncocytic thyroid adenomas, colon and prostate cancers [28,63-66]. Remarkably this control by SUMOylation is maintained despite the fact that usually, < 5% of target proteins are covalently modified (Figure 2A, for example).

SENP1 stimulates the transcriptional activity of ARs and two different mechanisms have been proposed. Cheng et al. [20] suggest that the transactivating effects of SENP1 do not involve SUMO deconjugation of the receptors, but rather cleavage of SUMO from HDAC1 thereby alleviated its repressive effect on AR activity. In contrast, Kaikkonen et al. [19] demonstrate that effects of SENP1 and SENP2 require intact SUMO acceptor sites in AR, indicating that the coactivating effects of the enzymes are directly on the receptors. We show here that both SENP1 and SENP2 stimulate the transcriptional activity of exogenous PR in HeLa cells (Figure 2B), and endogenous PR in T47Dco cells (Additional file 1: Figure S1B). This stimulatory effect is dependent on their enzymatic activity (Figure 2B), requires an intact PR SUMO conjugation site (Figure 3A, B), and functions only at promoters containing multiple PREs (Figure 3A, C). To test if SENP1 influences PR activity indirectly, we used the HDAC inhibitor TSA. Inhibition of HDAC activity by TSA did not prevent SENP1 stimulation of wild-type PR (Figure 8B). SUMOylation-deficient PR were similarly affected by TSA, indicating that other mechanisms are responsible for the suppressive effects of SUMOylation on PR activity. This is in agreement with a recent report showing that wild type and SUMOylation deficient AR are similarly influenced by TSA [19]. Taken together we conclude that SENPs target the PR SUMOylation site synergy control function.

### PR phosphorylation and SUMOylation

Both PR SUMOylation and PR phosphorylation are enhanced with similar kinetics by progestin binding to the receptors [18]. However, these two posttranslational protein modification steps appear to be independent of one another. We have shown that K388 SUMOylation of PRs, previously mutated at their MAPK-targeted, progestin-dependent Ser294/344/345 phosphorylation sites, is comparable to SUMOylation of wild-type PRs [6]. On the other hand, activation of MAPK signaling by overexpressing MEKK1 has complex, concentration-dependent effects on PR SUMOylation. At low concentrations, MEKK1 induces ligand-independent PR SUMOylation (Additional file 1: Figure S3B, lanes 3) and increases basal PR-dependent transcription (Figure 6). At high concentrations, MEKK1 suppresses hormone-dependent PR SUMOylation (Additional file 1: Figure S3B, lanes 6&8). These contrasting dual activities of MEKK1 suggest that the effects of MAPK on PR SUMOylation are indirect, through alteration of the activity of the general SUMOylation machinery. The molecular mechanisms by which MAPK signaling could indirectly influence PR SUMOylation include changes in the amounts and/or the activities of E3 ligases and cleaving enzymes [67,68]. In concert with our conclusions, Kaikkonen et al. [19] recently showed that AR phosphorylation has no effects on AR SUMOylation. Indeed, there are no phosphorylation-dependent SUMOylation motifs in either AR or PR. That PR phosphorylation at S294 does not affect PR SUMOylation is consistent with our data showing that there are no significant differences between the transcriptional activities of wild-type PR and an S294A PR mutant (Figure 6A, B). Qiu et al. [69] have shown similarly robust transcription with a PR S294A mutant. In contrast, Daniel et al. [18] concluded that an association does exist between hormone-dependent PR phosphorylation and PR SUMOylation. The reasons for these differences are unclear but may be related to experimental conditions including use of DNA concentrations for receptor expression at which squelching effects are observed [6].

In contrast to the stimulatory effects of SENP1 on PR activity (Figure 3A), the effect of MAPK signaling on PR transcriptional activity is not related directly to the deSUMOylase effect seen at high concentration (Additional file 1: Figure S3B). First, MEKK1 enhanced hormone independent PR activity (Figure 6A inset and Shen et al. [49]). Second, constitutively active NT-B cannot be SUMOylated, but can still be activated by MEKK1 (Additional file 1: Figure S3A). Third, although SUMOylation has no effect on the MMTV promoter (Figure 1C), MEKK enhances PR dependent activity on this promoter (data not shown). Taken together, our results suggest that the effects of MEKK do not depend on modulation of PR SUMOylation.

### Acetylation and SUMOylation

Acetylation of steroid receptors results in either transcriptional activation or repression, depending on alterations in DNA binding affinities, coregulator recruitment, or hormone responsiveness [16,70-72]. Acetylation and SUMOylation can in theory compete for the same Lys residue of some proteins [73]. In response to hormones, PRs are acetylated at a Lys-rich KxKK motif (aa 638-641) conserved in other steroid receptors, and located in the C-terminal hinge region [16]. However, for PR, a Lys to Arg mutation of these residues does not influence N-terminal SUMOylation [16]. We show that SENP1 does not influence the transcriptional activity of DBD-LBD (Figure 4B) which contains the acetylation motif (Figure 1A), suggesting dissociation between hinge region acetylation and deSUMOylation.

It has been suggested that SUMOylation represses transcription by recruiting repressors, including HDAC to SUMOylated substrates [46]. However, the transcriptional activities of wild-type and SUMOylation-deficient mutant PRs are both increased by the HDAC inhibitor TSA (Figure 8B), suggesting that other mechanisms are responsible for inhibition of PR activity by SUMOylation. Effects of TSA depend on the concentration used and the cell type analyzed [74,75]. Indeed, low concentrations (50 and 100 nM) of TSA enhance PR transcriptional activity (Figure 7A) as previously reported [76]. They also promote PR acetylation [16]. However, the effects of TSA on transcription are not related to receptor acetylation since an acetylation-deficient PR-B mutant retains heightened transcriptional activity [16]. On the other hand, at high concentrations (500 and 1000 nM) TSA markedly inhibits PR transcriptional activity (Figure 7A), and enhances protein stability (Figure 8A, lanes 9, 10). These results are in agreement with studies showing that TSA increases ER acetylation as well as protein stability without affecting ER transcript levels [71]. The inhibitory effect of high TSA levels on PR activity may in part be due to failed ligand dependent downregulation (Figure 8A; [49]), and in part to inhibition of coactivator expression and/or assembly. As we show in Figure 7C, overexpression of SRC1 relieves TSA inhibition in a dose dependent manner.

## Conclusions

PRs are major markers in breast cancer. Their presence indicates that a tumor is hormone-dependent and a candidate for endocrine therapies. The role of progesterone in activating these transcription factors is complex, however. After binding PR, progestin agonists and antagonists can have either transcriptional activating or suppressive effects modulated in part by enhancing or suppressing PR SUMOylation [6,18,31,33,35]. This study defines the roles of the SUMO-specific SENP proteases and SUMOylation on PR-dependent transcriptional synergy. 1. We show that deSUMOylation by SENP1 enhances transcriptional synergism in a promoter-specific manner. 2. We also show that SENPs, through their catalytic activity, act at the single K388 PR SUMOylation site, which if mutated eliminates transcriptional synergism by SENPs. 3. The enzymes can act only on hormone-bound full-length PRs and increase the ligand sensitivity of the receptors. 4. SUMOylation effects on PR transcriptional synergism are dissociable from receptor phosphorylation, SRC-1 coactivation or recruitment of HDACs to the promoter. We conclude that reversible SUMOylation/deSUMOylation of a minor PR protein subpopulation tightly controls the overall transcriptional activity of the receptors at complex synthetic promoters. Of note we previously showed a requirement for PR SUMOylation to transrepress ER thereby altering tumor responses to estrogens [33]. Taken together, our data suggest that the PR SUMO modification pathway critically modifies the response of a tumor to estrogens, progestins and antiprogestins--hormones that are major therapeutics for breast cancers.

## Methods

### Plasmids

The expression plasmids pSG5 hPR, encoding human PR-B and HEGO, encoding human ER, cloned into pSG5 were a gift of P. Chambon (Strasbourg, France). Cloning of pSG5 hPR1 K388R, pSG5 hPR1 S294/344/345A, pSG5 NT-B, pSG5 hPR1 R606A (PR-B DNA dimerization mutant was a gift of B. Jacobsen), pCMV5-MEKK1 and pSG5 DBD-LBD were described previously [4,11,17,33]. Wild type pEGFP-SUMO-1 was a gift of J. Palvimo and O. Janne (University of Helsinki, Helsinki, Finland). pCR3.1-SRC-1e was a gift of B. O'Malley (Baylor College of Medicine, Houston, TX). ERE_2_-Luc, PRE_2_-Luc and MMTV-Luc reporter plasmids were described previously [4]. Flag-SENP1, Flag-SENP1 mutant (R630L, K631M) and Flag-SENP2 were gifts of E. Yeh (M. D. Anderson, Houston, TX).

### Transcription assays

HeLa cells were plated in minimum Eagle's medium containing 5% FBS (twice charcoal-stripped for experiments with full-length PR or DBD-LBD) at a density of 1.2 × 10^5 ^cells per 60 mm dish, 1 day prior to transfection. Cells were transfected by calcium phosphate co-precipitation [42] with concentrations of expression vectors indicated in the figures. Reporter plasmids were added at 2 μg/dish. SV40-*Renilla *luciferase was added as an internal control at 20 ng/dish. Twenty four hours later, cells expressing LBD-containing constructs were washed and incubated 24 hrs with the synthetic progestin R5020 (Sigma Chemical Co., St. Louis, MO) at final concentrations indicated in the figures. Control cells received ethanol only. Cells were collected in 150 μl lysis buffer (Promega), and 50 μl were analyzed on a dual luminometer [42]. Results were normalized to Renilla luciferase activity and expressed as indicated in the figures. Replicate experiments were done in duplicate.

### Immunoblotting

Whole cell extracts were prepared from HeLa cells transiently transfected with PR expression vectors as described [33]. Cells were treated with 10 nM R5020 and/or Trichostatin A (TSA). Lysates containing equal protein concentrations were resolved by SDS-PAGE, transferred to nitrocellulose, and probed with anti-PR PgR1294 (DakoCytomation) or anti-β-actin AC-74 (Sigma) monoclonal antibodies. Bands were detected by enhanced chemiluminescence (PerkinElmer Life Sciences). For PR SUMOylation, HeLa cells cotransfected with PR and GFP-tagged SUMO-1 were collected in PBS containing 20 mM *N*-ethylmaleimide, and cell extracts were prepared in 50 mM Tris-HCl (pH 7.8), 150 mM NaCl, 5 mM EDTA, 15 mM dithiothreitol, a protease inhibitor mixture (Roche Molecular Biochemicals), and 20 mM *N*-ethylmaleimide. The expressed proteins were resolved on SDS-PAGE, and conjugated protein was detected by immunoblotting with PgR1294.

### Statistical analysis

Prism GraphPad software version 4 (GraphPad Software Inc. La Jolla, CA). was used to determine least-squares best fit of the experimental data to the theoretical dose-response curve. All values represent at least three independent experiments and are expressed as the means ± SD. Data sets were analyzed with GraphPad Prism 4 Statistical significance was determined by two-tailed unpaired student's *t *test, and the differences were considered statistically significant at a P value of 0.05.

## Abbreviations

AF: Activation function; AR: Androgen receptors; BUS: PR-B-upstream segment; DBD: DNA binding domain; ER: Estrogen receptors; GFP: Green fluorescent protein; GR: Glucocorticoid receptors; HDAC: Histone deacetylase; LBD: Ligand binding domain; MMTV: Mouse mammary tumor virus; MR: Mineralocorticoid receptors; NEM: *N*-ethylmaleimide; NR: Nuclear receptors; NT-B: N-terminal region of PR-B; PIAS: Protein inhibitor of activated transducer and activator of transcription; PR: Progesterone receptors; PRE: Progesterone response element; SENP: SUMO specific protease; SRC: Steroid receptor coactivator; SUMO: Small ubiquitin like modifier; TSA: Trichostatin A

## Competing interests

The authors declare that they have no competing interests.

## Authors' contributions

HA-H was involved in all of the experimental and theoretical work. HA-H and KBH participated in the design of the experiments. HA-H and KBH wrote the manuscript. All authors have read and approved the final manuscript.

## Supplementary Material

Additional file 1 **Figure S1. A) DeSUMOylation of PR by SENP1 depends on its catalytic activity**. HeLa cells were transiently transfected with expression vectors encoding wild type PR-B together with a GFP-SUMO-1 expression vector (+), and wild type or mutant (m) SENP1. Cells were treated 24 hrs without (-) or with (+) 10 nM R5020. Western blot analysis was performed on cell extracts probed with the anti-PR1294 monoclonal antibody or anti -actin control. **B) SENP1 enhances PR-B activity in T47D breast cancer cells**. PR-negative T47D-Y breast cancer cells stably expressing PR-B were transfected with the PRE2-Luc reporter plasmid in the presence of pSV40-Renilla as internal control along with increasing amount (20-1000 ng) of SENP1 expression vector, or an empty vector control (-). Cells were treated without (-) or with (+) 10 nM R5020 for 24 hrs before being assayed for luciferase activity. **C) SENP1 enhances transcription by the partial agonist RU486**. HeLa cells were transfected with 2 g of PRE2-luciferase reporters together with 50 ng of a PR-B expression vector and Renilla-Luc as an internal control in the presence or absence of 100 ng SENP1 or SENP1m expression vectors. The cells were treated for 24 hrs with the agonist R5020 (10 nM), partial agonist RU486 (100 nM), or the pure antagonist ZK98299 (100 nM) then harvested and lysed. The extracts were assayed for luciferase activities as in Figure 1. **Figure S2. The PR DBD dimerization interface is necessary for effective synergy control**. HeLa cells were transfected with 2 g of PRE2-luciferase reporters together with 50 ng of a wild type PR -B, the PR-B K388R SUMOylation deficient, or a PR-B DBD dimerization mutant (PR-B DX) expression vector and Renilla-Luc as an internal control in the presence or absence of 100 ng SENP1 expression vectors. The cells were treated for 24 hrs with the agonist R5020 (10 nM), then harvested and lysed. The extracts were assayed for luciferase activities as in Figure 1. **Figure S3. A**) **The stimulatory effect of MEKK1 on PR-B transcriptional activity is LBD and hormone independent**. HeLa cells were transfected with 2 g of PRE2-luciferase reporters together with 500 ng of NTB-DBD, a constitutively active PR N-terminal expression vector in the presence of pSV40-Renilla as internal control along with increasing amount (5-200 ng) of constitutively active MEKK1 expression vector, or an empty vector control (-). The extracts were assayed for luciferase activities as in Figure 1. **B) Concentration dependent effect of MEKK1 on PR SUMOylation**. HeLa cells were transiently transfected with expression vectors encoding wild type PR-B together with a GFP-SUMO-1 expression vector (+) in the absence (-) or presence of increasing amount of MEKK1 expression vector. Cells were treated 24 hrs without (-) or with (+) 10 nM R5020. Western blot analysis was performed on cell extracts probed with the anti-PR1294 monoclonal antibody or anti -actin control. Click here for file
