# Supplementary material for: Control of progesterone receptor transcriptional synergy by SUMOylation and deSUMOylation
Source: BMC Mol Biol. 2012 Mar 22;13:10. doi: 10.1186/1471-2199-13-10 (PMC3373386; doi:10.1186/1471-2199-13-10)
Supplement: Additional file 1 — Figure S1. A) DeSUMOylation of PR by SENP1 depends on its catalytic activity. HeLa cells were transiently transfected with expression vectors encoding wild type PR-B together with a GFP-SUMO-1 expression vector (+), and wild type or mutant (m) SENP1. Cells were treated 24 hrs without (-) or with (+) 10 nM R5020. Western blot analysis was performed on cell extracts probed with the anti-PR1294 monoclonal antibody or anti -actin control. B) SENP1 enhances PR-B activity in T47D breast cancer cells. PR-negative T47D-Y breast cancer cells stably expressing PR-B were transfected with the PRE2-Luc reporter plasmid in the presence of pSV40-Renilla as internal control along with increasing amount (20-1000 ng) of SENP1 expression vector, or an empty vector control (-). Cells were treated without (-) or with (+) 10 nM R5020 for 24 hrs before being assayed for luciferase activity. C) SENP1 enhances transcription by the partial agonist RU486. HeLa cells were transfected with 2 g of PRE2-luciferase reporters together with 50 ng of a PR-B expression vector and Renilla-Luc as an internal control in the presence or absence of 100 ng SENP1 or SENP1m expression vectors. The cells were treated for 24 hrs with the agonist R5020 (10 nM), partial agonist RU486 (100 nM), or the pure antagonist ZK98299 (100 nM) then harvested and lysed. The extracts were assayed for luciferase activities as in Figure 1. Figure S2. The PR DBD dimerization interface is necessary for effective synergy control. HeLa cells were transfected with 2 g of PRE2-luciferase reporters together with 50 ng of a wild type PR -B, the PR-B K388R SUMOylation deficient, or a PR-B DBD dimerization mutant (PR-B DX) expression vector and Renilla-Luc as an internal control in the presence or absence of 100 ng SENP1 expression vectors. The cells were treated for 24 hrs with the agonist R5020 (10 nM), then harvested and lysed. The extracts were assayed for luciferase activities as in Figure 1. Figure S3. A) The stimulatory effect of [file 1471-2199-13-10-S1.PDF]

Figure 1S

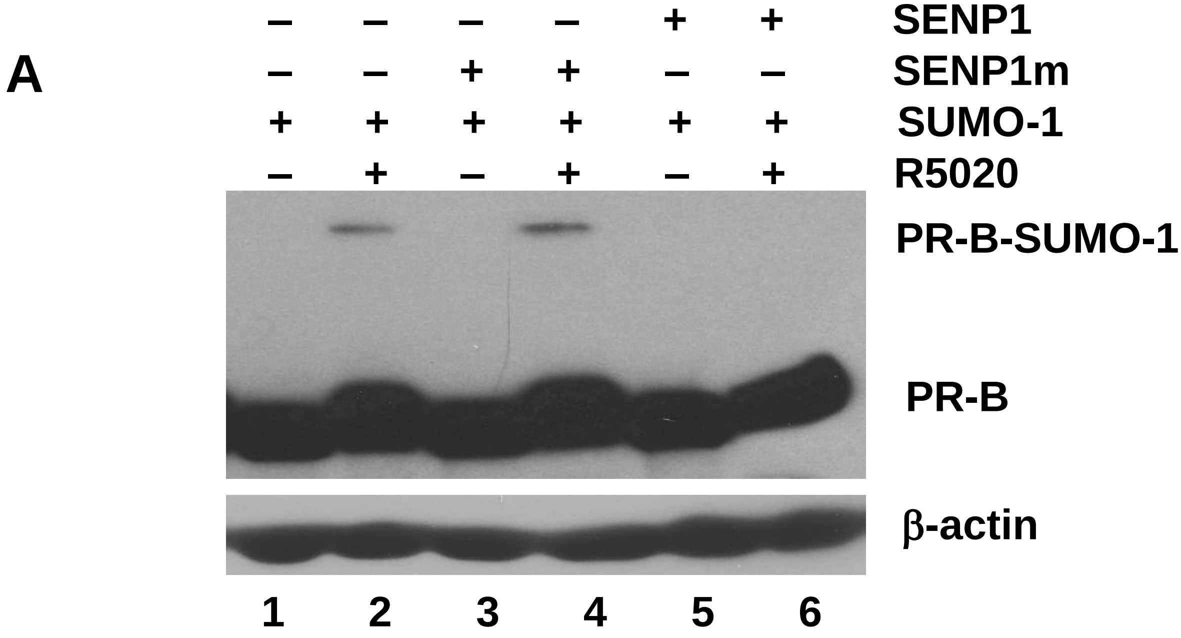
A
– – – – + + SENP1
– – + + – – SENP1m
+ + + + + + SUMO-1
– + – + – + R5020
PR-B-SUMO-1
PR-B
β-actin
1 2 3 4 5 6

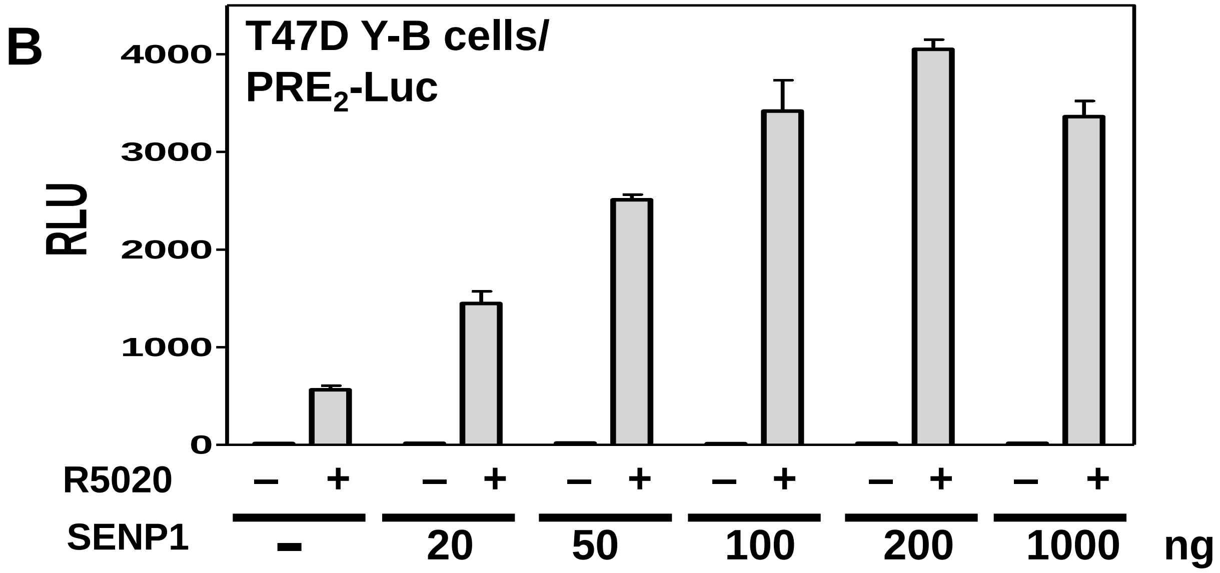
B
T47D Y-B cells/
PRE2-Luc
RLU
4000
3000
2000
1000
0
R5020
– + – + – + – + – + – +
SENP1
– 20 50 100 200 1000 ng

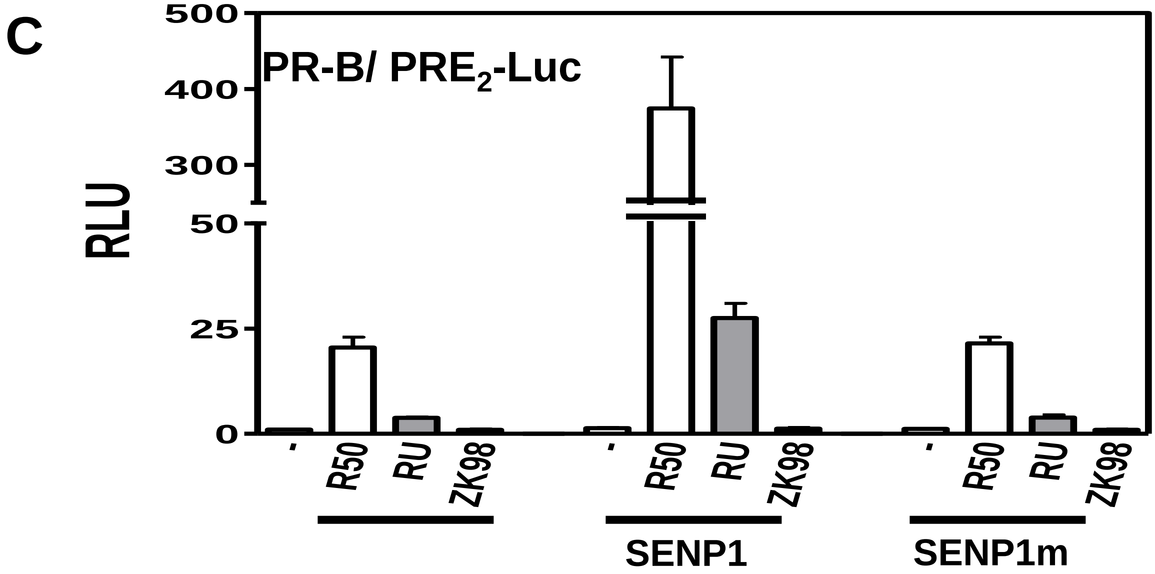
C
PR-B/ PRE2-Luc
RLU
500
400
300
50
25
0
- R50 RU ZK98
- R50 RU ZK98
- R50 RU ZK98
SENP1
SENP1m

Figure 2S

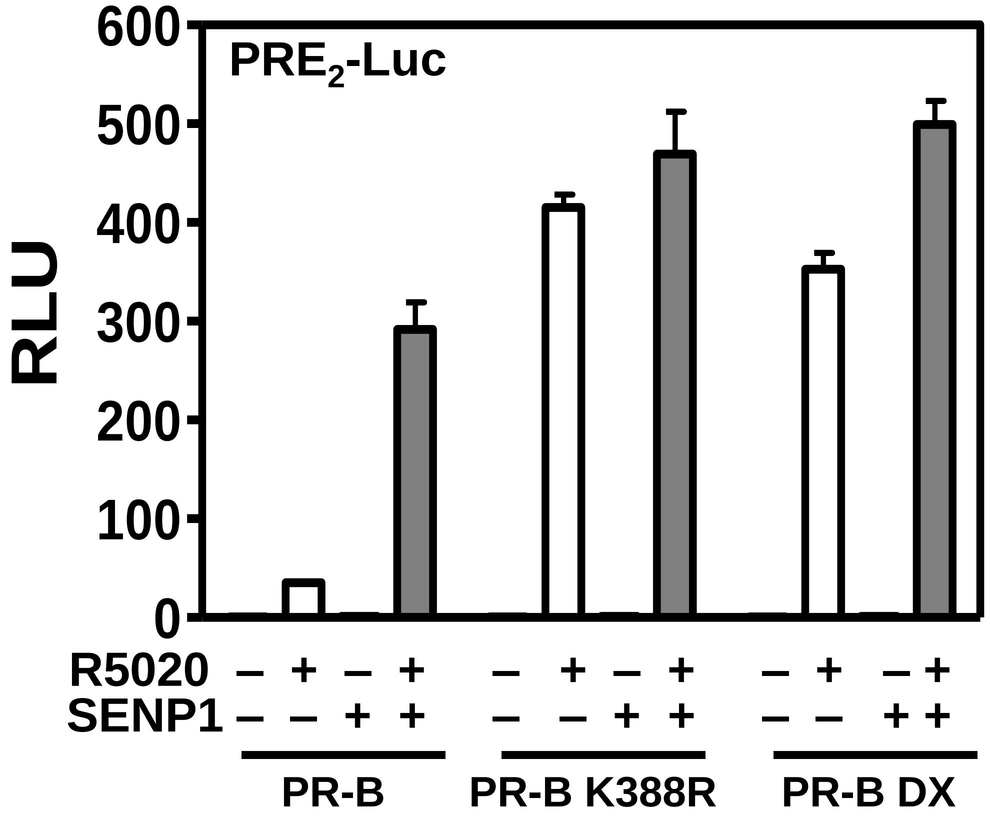

PRE2-Luc
RLU
600
500
400
300
200
100
0
R5020 – + – + – + – + – + – +
SENP1 – – + + – – + + – – + +
PR-B
PR-B K388R
PR-B DX

Figure 3S

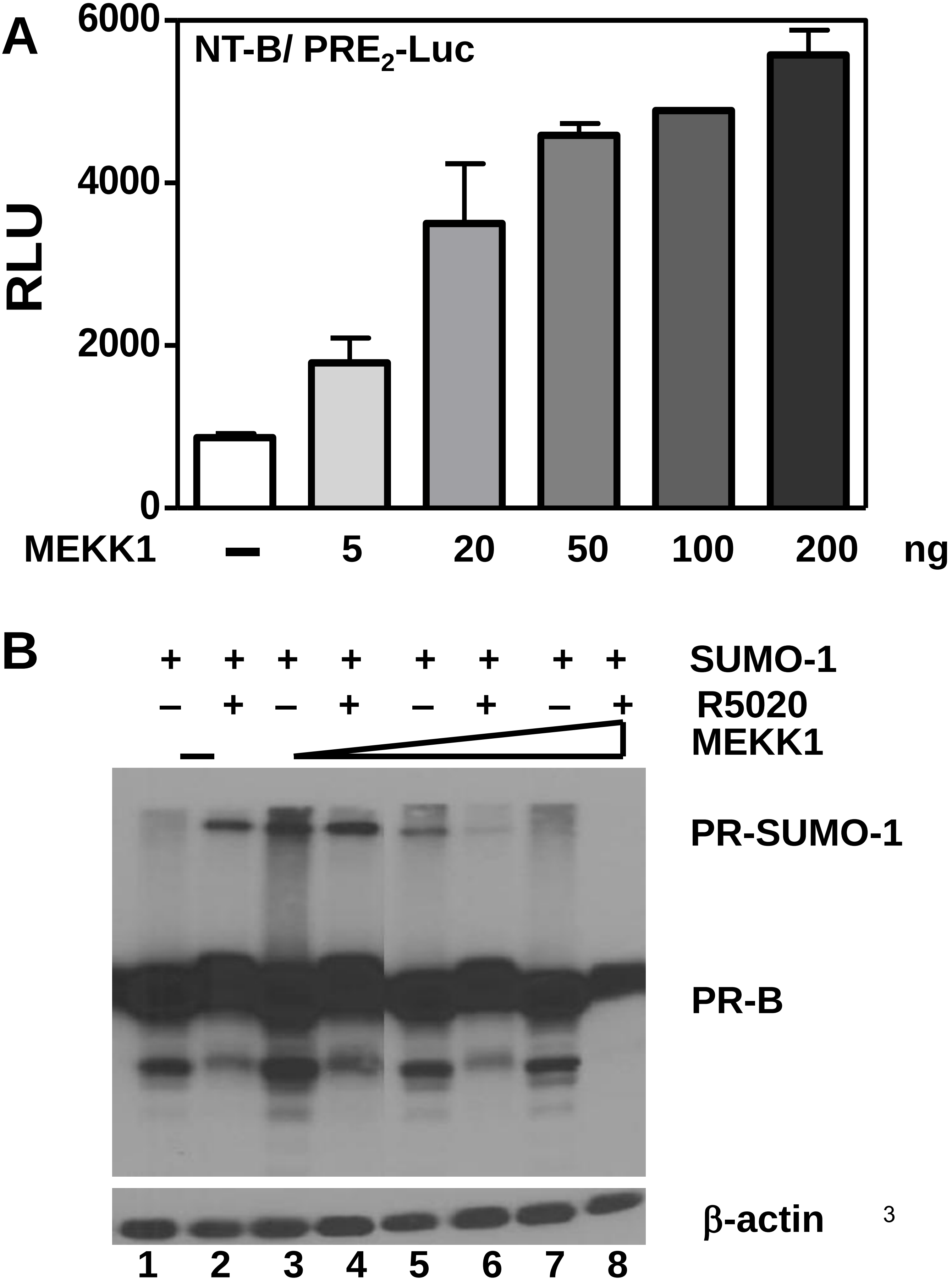

A
NT-B/ PRE$_2$-Luc
RLU
6000
4000
2000
0
MEKK1
–
5
20
50
100
200
ng
B
SUMO-1
R5020
MEKK1
PR-SUMO-1
PR-B
β-actin
1
2
3
4
5
6
7
8

Supplemental Figure Legends

Supplemental Figure 1S. A) DeSUMOylation of PR by SENP1 depends on its catalytic activity. HeLa cells were transiently transfected with expression vectors encoding wild type PR-B together with a GFP-SUMO-1 expression vector (+), and wild type or mutant (m) SENP1. Cells were treated 24 hrs without (-) or with (+) 10 nM R5020. Western blot analysis was performed on cell extracts probed with the anti-PR1294 monoclonal antibody or anti β-actin control. **B) SENP1 enhances PR-B activity in T47D breast cancer cells.** PR-negative T47D-Y breast cancer cells stably expressing PR-B were transfected with the PRE_2-Luc reporter plasmid in the presence of pSV40-Renilla as internal control along with increasing amount (20-1000 ng) of SENP1 expression vector, or an empty vector control (-). Cells were treated without (-) or with (+) 10 nM R5020 for 24 hrs before being assayed for luciferase activity. **C) SENP1 enhances transcription by the partial agonist RU486.** HeLa cells were transfected with 2 μg of PRE_2-luciferase reporters together with 50 ng of a PR-B expression vector and Renilla-Luc as an internal control in the presence or absence of 100ng SENP1 or SENP1m expression vectors. The cells were treated for 24 hrs with the agonist R5020 (10 nM), partial agonist RU486 (100 nM), or the pure antagonist ZK98299 (100 nM) then harvested and lysed. The extracts were assayed for luciferase activities as in Figure 1.

Supplemental Figure 2S. The PR DBD dimerization interface is necessary for effective synergy control. HeLa cells were transfected with 2 μg of PRE_2-luciferase reporters together with 50 ng of a wild type PR-B, the PR-B K388R SUMOylation deficient, or a PR-B DBD dimerization mutant (PR-B DX) expression vector and Renilla-Luc as an internal control in the presence or absence of 100 ng SENP1 expression vectors. The cells were treated for 24 hrs with the agonist R5020 (10 nM), then harvested and lysed. The extracts were assayed for luciferase activities as in Figure 1.

Supplemental Figure 3S. A) The stimulatory effect of MEKK1 on PR-B transcriptional activity is LBD and hormone independent. HeLa cells were transfected with 2 μg of PRE_2-luciferase reporters together with 500 ng of NTB-DBD, a constitutively active PR N-terminal expression vector in the presence of pSV40-Renilla as internal control along with increasing amount (5-200 ng) of constitutively active MEKK1 expression vector, or an empty vector control (-). The extracts were assayed for luciferase activities as in figure 1. **B) Concentration dependent effect of MEKK1 on PR SUMOylation.** HeLa cells were transiently transfected with expression vectors encoding wild type PR-B together with a GFP-SUMO-1 expression vector (+) in the absence (-) or presence of increasing amount of MEKK1 expression vector. Cells were treated 24 hrs without (-) or with (+) 10 nM R5020. Western blot analysis was performed on cell extracts probed with the anti-PR1294 monoclonal antibody or anti β-actin control.
